# Nomogram for predicting the overall survival and cancer-specific survival of patients with intraductal carcinoma of the prostate

**DOI:** 10.1007/s00432-023-05582-5

**Published:** 2024-01-28

**Authors:** Yongqiang Cui, Junyang Lin, Dingqi Sun, Hui Zhang, Tongxiang Diao, Qiang Fu

**Affiliations:** 1 Department of Urology, Shandong Provincial Hospital Affiliated to Shandong First Medical University, 324 Jingwuweiqi Rd, Jinan, 250021 Shandong China; 2 Department of Urology, Shandong Provincial Hospital, Shandong University, Jinan, 250021 China; 3 Key Laboratory of Urinary Diseases in Universities of Shandong Shandong First Medical University, Jinan, 250021 China

**Keywords:** Prostate cancer, Infiltrating ductal carcinoma, SEER database, Nomogram, Prognosis

## Abstract

**Purpose:**

Intraductal carcinoma of the prostate (IDC-P) is a histological subtype that differs from conventional acinar adenocarcinoma in terms of its origin, appearance, and pathological features. For IDC-P, there is currently no recognized best course of action, and its prognosis is unclear. The goal of this study is to analyze independent prognostic factors in IDC-P patients and to develop and validate a nomogram to predict overall survival (OS) and cancer-specific survival (CSS).

**Methods:**

Clinical data for IDC-P patients were collected from the Surveillance, Epidemiology, and End Results database. To identify the independent variables influencing prognosis, multivariate Cox regression analysis was performed. A nomogram model was created utilizing these variables after comparing the variations in OS and CSS among various subgroups using Kaplan‒Meier curves. Internal validation of the nomograms was verified using the bootstrap resampling method.

**Results:**

The study included 280 IDC-P patients in total. Marital status, summary stage, grade, and the presence of lung metastases were significant factors impacting OS, and CSS was significantly influenced by marital status, summary stage, AJCC stage, the presence of lung metastases, the presence of bone metastases, and PSA according to univariate and multivariate Cox regression models (*P < *0.05). Nomogram models were created to estimate OS and CSS using these parameters. The OS prediction model’s C-index was 0.744, whereas the CSS prediction model’s C-index was 0.831.

**Conclusion:**

We developed and verified nomogram models for the prediction of 1-, 3-, and 5-year OS and CSS in patients with IDC-P. These nomograms serve as a resource for evaluating patient prognosis, therapy, and diagnosis, ultimately improving clinical decision-making accuracy.

## Introduction

One of the most prevalent malignant tumors in men today, particularly in older men, is prostate cancer. The survival of cancer patients is increasingly threatened by its progression. There are significant regional and racial differences in the incidence of prostate cancer, with rates as high as 21.4% in industrialized nations like Europe, America, and the Caribbean and rates as low as 1.6% in Asian regions like China, South Korea, and Japan (Torre et al. 2016; Suzuki et al. 2010). The ductal glandular cells of the prostate are the source of IDC-P. It is typically detected in people over 50 years old and has a relatively low overall incidence, accounting for 0.5–6% of all diagnosed instances of prostate cancer (Dube et al. 1973; Greene et al. 1979). IDC-P was categorized as a separate pathological entity from other types of prostate cancer in the 2016 WHO pathological classification (Moch et al. 2016). The proliferation of cancerous cells in the prostatic ducts and acini is a feature of IDC-P (Szentirmai and Giannico 2020). PSA analysis and needle biopsy are often necessary for the diagnosis of IDC-P. If the pathology is positive, additional imaging tests such MRIs, bone scans, and ultrasounds can be utilized to determine how far the tumor has spread. The influence of IDC-P on the prognosis of prostate cancer patients has gained more attention as our understanding of the histology of the disease has grown. IDC-P has been proven to significantly affect patient prognosis, with malignancy increasing with tumor volume and substantial associations with metastasis and the development of prostate cancer. IDC-P has molecular and immunohistochemical features of an invasive tumor (Samaratunga et al. 2020; Gandhi et al. 2020). Research on the effects of IDC-P on prostate cancer progression and prognosis is now largely restricted to non-metastatic prostate cancer, and the findings is still somewhat debatable. The clinical importance of IDC-P has received more focus in recent years. The clinical data currently available suggest that the presence of IDC-P is strongly correlated with a poor outcome in patients with both localized and advanced stages of prostate cancer (Lawrence et al. 2020). However, there are still several contentious issues surrounding IDC-P that have not been satisfactorily addressed, both at the morphological and molecular levels, and there are no effective therapeutic strategies that directly target IDC-P. The work by Meeks et al. (2012) revealed the characteristics of prostate intraductal cancer; however, more research on prognostic variables is needed. It is critical to improve the prognosis of IDC-P. Therefore, it is crucial to investigate the clinical, pathological, demographic, and prognostic aspects of IDC-P in a sizable population.

In the United States, the Surveillance, Epidemiology, and End Results (SEER) database is a reliable source of clinical data on cancer. It gathers information on the demographic traits, disease conditions, therapeutic options, and fundamental prognostic data of cancer patients. Large datasets are available in the SEER database, which supports thorough clinical research. Nomograms can be used as clinical prediction models to forecast the likelihood that certain patients will experience endpoint events. Most variables in a nomogram have been identified through multivariable Cox regression. In the current literature, nomograms have been generated and shown to be helpful in guiding the treatment of a variety of malignancies. The large sample size in the publicly accessible SEER database will be used in this study to fully and methodically investigate the clinical characteristics and prognosis of and risk factors for intraductal IDC-P. The project will create and verify a nomogram prediction model, explore the survival benefits and independent determinants of survival benefits for IDC-P patients, and offer clinical advice.

### Data selection and inclusion criteria

The National Cancer Institute SEER database was used in this retrospective cohort analysis. From the SEER database, we chose all cases of prostate cancer having the ICD-O-3 code 8500/3: infiltrating duct carcinoma.

First-time diagnoses between January 2010 and December 2015, a pathological confirmation of primary prostate cancer, the availability of complete general clinical and follow-up data, and an ICD-O-3 code for infiltrating duct carcinoma were the inclusion criteria. Patients who were not the first malignant primary indicator, patients without a histological diagnosis, and patients with insufficient data were excluded from the study. Race, marital status, age, vital status, median household income, year of diagnosis, PSA, grade, Gleason score, summary stage, AJCC stage, TNM stage, bone metastases, brain metastases, liver metastases, lung metastases, surgery type, removed lymph nodes, radiation, chemotherapy, and systemic therapy data were all retrieved. OS and tumor-specific survival at 1, 3, and 5 years were the study’s main objectives.

Using Seer*Stat8.4.0.1, the information was filtered and gathered. The data of this research were obtained from public databases, and no ethical approval was needed.

### Clinical and demographic characteristics

The following demographic factors are primarily examined in this study: race (Black, White, Other (Asian/Pacific Islander/American Indian/Alaska Native)), marital status (single, married, divorced, other), age (years) (≤ 60, 61–70, 71–80, > 80), median household income (< $60, 000, ≥ $60, 000), year of diagnosis (2010, 2011, 2012, 2013, 2014, 2015), PSA(0–3.9 ng/ml, 4–10 ng/ml, 10.1–20 ng/ml, > 20 ng/ml), grade (poorly differentiated, well differentiated, and moderately differentiated), Gleason score (< 7, 7, > 7), summary stage (localized, regional, distant), AJCC stage (I and II, III and IV), T stage (T1, T2, T3, T4), N stage (N0, N1), M stage (M0, M1), bone metastases (no, yes), brain metastases (no, yes), liver metastases (no, yes), lung metastases (no, yes), surgery type (no surgery, TURP, radical prostatectomy), lymph nodes removed (none, 1 to 3 regional lymph nodes removed, 4 or more regional lymph nodes removed), radiation (no/unknown, yes), chemotherapy (no/unknown, yes), systemic therapy (no, yes), and vital status.

### Statistical analysis

The endpoints in this study were OS and CSS. The clinical and pathological features of the patients who were included were detailed. To find independent factors affecting OS or CSS, univariate and multivariate Cox regression analyses were conducted, with a significance threshold of *P < *0.05 being deemed statistically significant. As hazard ratios (HRs) with 95% confidence intervals (CIs), the data are displayed. The Kaplan‒Meier method was used to create patient survival curves, and the log-rank test was applied to assess disparities in survival and examine how these factors affected prognosis. Nomograms were created to predict 1-, 3-, and 5-year OS and CSS in IDC-P patients using significant variables found in the multivariate Cox analysis. One thousand bootstrap resamples were used to plot calibration curves for model validation. Statistical analyses were completed using R version 4.3.0 and SPSS 26 software. *P* values < 0.05 were considered indicative of statistical significance.

## Results

### Patient clinical characteristics

The study comprised 280 patients in total from the SEER database. Patients were predominantly White and between the ages of 61 and 70 most frequently. The majority of patients had PSA levels between 4 and 10 ng/ml. Over half of the patients had a Gleason score greater than 7, and the grade distribution was mainly in the poorly differentiated category. The majority of patients did not have lymph node metastases or distant metastasis, and the T stage was primarily localized between T2 and T3. Radiation therapy was administered to 66.1% of patients, chemotherapy to 96.1% of patients, and radical prostatectomy to 58.9% of patients. A total of 54.6% of patients did not have lymph node clearance. In addition, systemic therapy was given to 77.5% of patients. Clinical characteristics of the study population are given in Table 1.

**Table 1:** Patient characteristics of IDC-P

Variables	*n*	%
Race		
Black	39	13.9
White	219	78.2
Other(Asian/Pacific Islander/American Indian/Alaska Native)	22	7.9
Marital status		
Single	23	8.2
Married	197	70.4
Divorced	23	8.2
Other	37	13.2
Age (years)		
≤ 60	79	28.2
61–70	104	37.1
71–80	77	27.5
> 80	20	7.1
Vital status		
Alive	203	72.5
Dead	77	27.5
Median household income		
< $60,000	99	35.4
≥ $60,000	181	64.6
Year of diagnosis		
2010	54	19.3
2011	47	16.8
2012	46	16.4
2013	35	12.5
2014	49	17.5
2015	49	17.5
PSA(ng/ml)		
0–3.9	38	13.6
4–10	149	53.2
10.1–20	37	13.2
> 20	56	20
Grade		
Poorly differentiated	235	83.9
Well differentiated and moderately differentiated	45	16.1
Gleason score		
< 7	29	10.4
7	84	30
> 7	167	59.6
Summary stage		
Localized	131	46.8
Regional	120	42.9
Distant	29	10.4
AJCC stage		
I and II	132	47.1
III and IV	148	52.9
T stage		
T1	46	16.4
T2	106	37.9
T3	105	37.5
T4	23	8.2
N stage		
N0	249	88.9
N1	31	11.1
M stage		
M0	251	89.6
M1	29	10.4
Bone metastases		
No	256	91.4
Yes	24	8.6
Brain metastases		
No	280	100
Liver metastases		
No	280	100
Lung metastases		
No	276	98.6
Yes	4	1.4
Surgery type		
No surgery	76	27.1
TURP	39	13.9
Radical prostatectomy	165	58.9
Lymph nodes removed		
None	153	54.6
1–3 regional lymph nodes removed	24	8.6
4 or more regional lymph nodes removed	103	36.8
Radiation		
No/unknown	185	66.1
Yes	95	33.9
Chemotherapy		
No/unknown	269	96.1
Yes	11	3.9
Systemic therapy		
No	217	77.5
Yes	63	22.5

### Selection of prognostic factors

Marital status, summary stage, grade, and the presence of lung metastases were significant factors impacting OS prognosis in multivariate Cox regression analysis (*P < *0.05). The following relevant factors were found to significantly alter the prognosis for CSS: marital status, summary stage, AJCC stage, the presence of lung metastases, the presence of bone metastases, and PSA (*P < *0.05). On the other hand, age, Gleason score, and race were not discovered to be important predictive factors. (Table 2).

**Table 2:** Univariate and multivariate Cox analysis of prognosis factors of IDC-P on OS and CSS

Variables	Univariate		Multivariate		Univariate		Multivariate	
	Overall survival				Cancer-specific survival			
	Univariate		Multivariate		Univariate		Multivariate	
	HR	*P*	HR	*P*	HR	*P*	HR	*P*
Race								
Black								
White	0.934 (0.501–1.740)	0.829			1.057 (0.442–2.530)	0.901		
Other (Asian/Pacific Islander/American Indian/Alaska Native)	0.623 (0.200–1.935)	0.413			0.946 (0.235–3.804)	0.937		
Marital status								
Single								0.041
Married	0.427 (0.214–0.853)	0.016	0.337 (0.156–0.728)	0.006	0.347 (0.149–0.810)	0.014	0.209 (0.068–0.649)	0.007
Divorced	0.993 (0.412–2.394)	0.987	0.562 (0.202–1.564)	0.27	0.980 (0.341–2.812)	0.969	0.417 (0.099–1.747)	0.231
Other	0.901 (0.405–2.005)	0.798	0.513 (0.207–1.271)	0.149	0.525 (0.176–1.563)	0.247	0.196 (0.046–0.834)	0.027
Age (years)								
≤ 60								0.109
61–70	1.655 (0.831–3.297)	0.152	1.735 (0.794–3.793)	0.167	1.677 (0.723–3.892)	0.229	4.146 (1.136–15.140)	0.031
71–80	2.725 (1.372–5.410)	0.004	2.651 (1.152–6.103)	0.022	1.663 (0.668–4.141)	0.275	4.729 (1.119–19.981)	0.035
> 80	6.517 (2.956–14.365)	0	3.123 (1.205–8.096)	0.019	4.988 (1.797–13.845)	0.002	6.352 (1.226–32.910)	0.028
Median household income								
< $60,000								
≥ $60,000	0.640 (0.408–1.005)	0.053			0.512 (0.282–0.932)	0.028	0.356 (0.168–0.754)	0.007
Year of diagnosis								
2010								
2011	0.955 (0.467–1.953)	0.9			0.964 (0.350–2.659)	0.944		
2012	1.480 (0.731–2.998)	0.276			1.412 (0.544–3.666)	0.478		
2013	1.453 (0.661–3.197)	0.353			1.518 (0.547–4.208)	0.423		
2014	0.975 (0.421–2.255)	0.952			1.073 (0.369–3.125)	0.897		
2015	0.930 (0.387–2.235)	0.871			0.931 (0.300–2.893)	0.902		
PSA (ng/ml)								
0–3.9								0.04
4–10	0.486 (0.253–0.933)	0.03	0.588 (0.295–1.171)	0.131	0.348 (0.144–0.843)	0.019	0.349 (0.117–1.038)	0.058
10.1–20	0.635 (0.263–1.535)	0.313	0.745 (0.287–1.936)	0.546	0.488 (0.147–1.622)	0.242	0.593 (0.142–2.480)	0.474
> 20	1.682 (0.859–3.294)	0.13	1.469 (0.683–3.163)	0.325	1.872 (0.812–4.318)	0.141	1.576 (0.536–4.638)	0.409
Grade								
Poorly differentiated								
Well differentiated and moderately differentiated	0.068 (0.009–0.491)	0.008	0.114 (0.014–0.920)	0.041	0.038 (0.001–1.226)	0.065		
Gleason score								
< 7								0.35
7	2.166 (0.484–9.687)	0.312	0.890 (0.172–4.601)	0.889	0.683 (0.125–3.734)	0.66	0.202 (0.023–1.796)	0.151
> 7	6.619 (1.616–27.109)	0.009	1.022 (0.206–5.057)	0.979	3.700 (0.890–15.383)	0.072	0.367 (0.056–2.404)	0.296
Summary stage								
Localized								0.003
Regional	1.380 (0.816–2.334)	0.23	21.026 (1.922–229.959)	0.013	2.632 (1.144–6.056)	0.023	220.479 (9.217–5274.144)	0.001
Distant	8.062 (4.431–14.669)	0	11.844 (0.677–207.176)	0.09	20.719 (8.816–48.696)	0	83.976 (2.081–3388.968)	0.019
AJCC stage								
I and II								
III and IV	1.927 (1.203–3.085)	0.006	0.085 (0.007–1.094)	0.059	3.900 (1.869–8.137)	0	0.014 (0.001–0.344)	0.009
T stage								
T1								0.414
T2	0.364 (0.19–0.695)	0.002	0.618 (0.296–1.293)	0.202	0.472 (0.175–1.269)	0.137	1.285 (0.362–4.564)	0.698
T3	0.447 (0.239–0.834)	0.011	0.674 (0.210–2.164)	0.507	0.704 (0.281–1.765)	0.454	0.939 (0.177–4.980)	0.941
T4	2.949 (1.504–5.782)	0.002	1.299 (0.491–3.439)	0.599	5.790 (2.315–14.481)	0	2.218 (0.562–8.750)	0.255
N stage								
N0								
N1	1.742 (0.917–3.309)	0.09			3.350 (1.686–6.659)	0.001	2.512 (0.840–7.514)	0.099
M stage								
M0								
M1	6.855 (4.050–11.602)	0	11.844 (0.677–207.176)	0.09	11.790 (6.285–22.116)	0		
Bone metastases								
No								
Yes	8.388 (4.827–14.578)	0	3.928 (0.803–19.213)	0.091	13.100 (6.838–25.096)	0	7.732 (1.007–59.377)	0.049
Lung metastases								
No								
Yes	13.545 (4.743–38.681)	0	3.926 (1.105–13.954)	0.035	22.460 (7.581–66.546)	0	8.485 (1.856–38.791)	0.006
Surgery type								
No surgery								0.159
TURP	1.700 (0.974–2.967)	0.062	0.616 (0.248–1.532)	0.298	2.770 (1.338–5.734)	0.006	0.740 (0.187–2.931)	0.669
Radical prostatectomy	0.278 (0.162–0.476)	0	0.188 (0.040–0.871)	0.033	0.354 (0.164–0.764)	0.008	0.052 (0.003–1.068)	0.055
Lymph nodes removed								
None								0.258
1–3 regional lymph nodes removed	0.451 (0.180–1.129)	0.089	2.838 (0.536–15.027)	0.22	0.328 (0.078–1.374)	0.127	13.374 (0.576–310.47)	0.106
4 or more regional lymph nodes removed	0.378 (0.216–0.659)	0.001	1.950 (0.456–8.341)	0.368	0.422 (0.207–0.860)	0.018	8.279 (0.546–125.493)	0.128
Radiation								
No/unknown								
Yes	1.282 (0.806–2.038)	0.294			1.464 (0.798–2.686)	0.218		
Chemotherapy								
No/unknown								
Yes	1.976 (0.720–5.427)	0.186			3.435 (1.223–9.647)	0.019	1.179 (0.316–4.404)	0.806
Systemic therapy								
No								
Yes	2.464 (1.547–3.924)	0	1.775 (0.814–3.869)	0.149	3.971 (2.178–7.243)	0	2.500 (0.791–7.900)	0.118

### Survival analysis

**Fig. 1:**
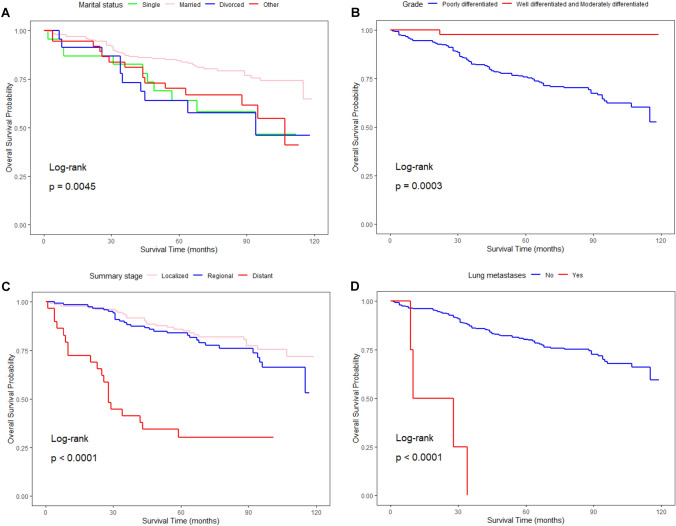
The Kaplan‒Meier survival curves show the different effects of different clinical parameters on the OS rate of IDC-P patients: **A** marital status (*P = *0.0045), **B** grade (*P = *0.0003), **C** summary stage (*P < *0.0001), **D** lung metastases (*P < *0.0001)

**Fig. 2:**
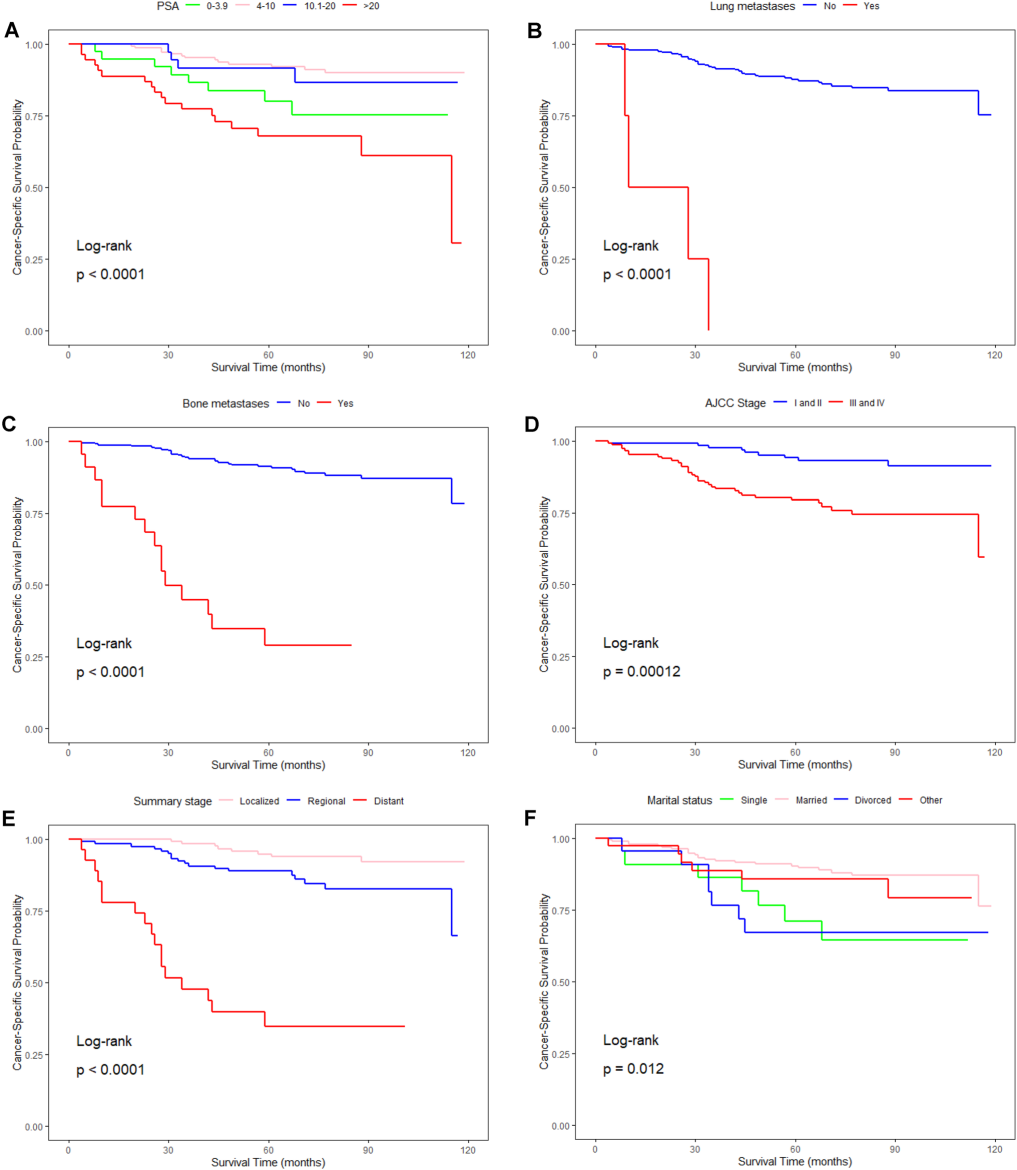
The Kaplan‒Meier survival curves show the different effects of different clinical parameters on the CSS rate of IDC-P patients: **A** PSA (*P < *0.0001), **B** lung metastases (*P < *0.0001), **C** bone metastases (*P < *0.0001), **D** AJCC stage (*P = *0.00012), **E** summary stage(*P < *0.0001), **F** marital status (*P = *0.012)

**Fig. 3:**
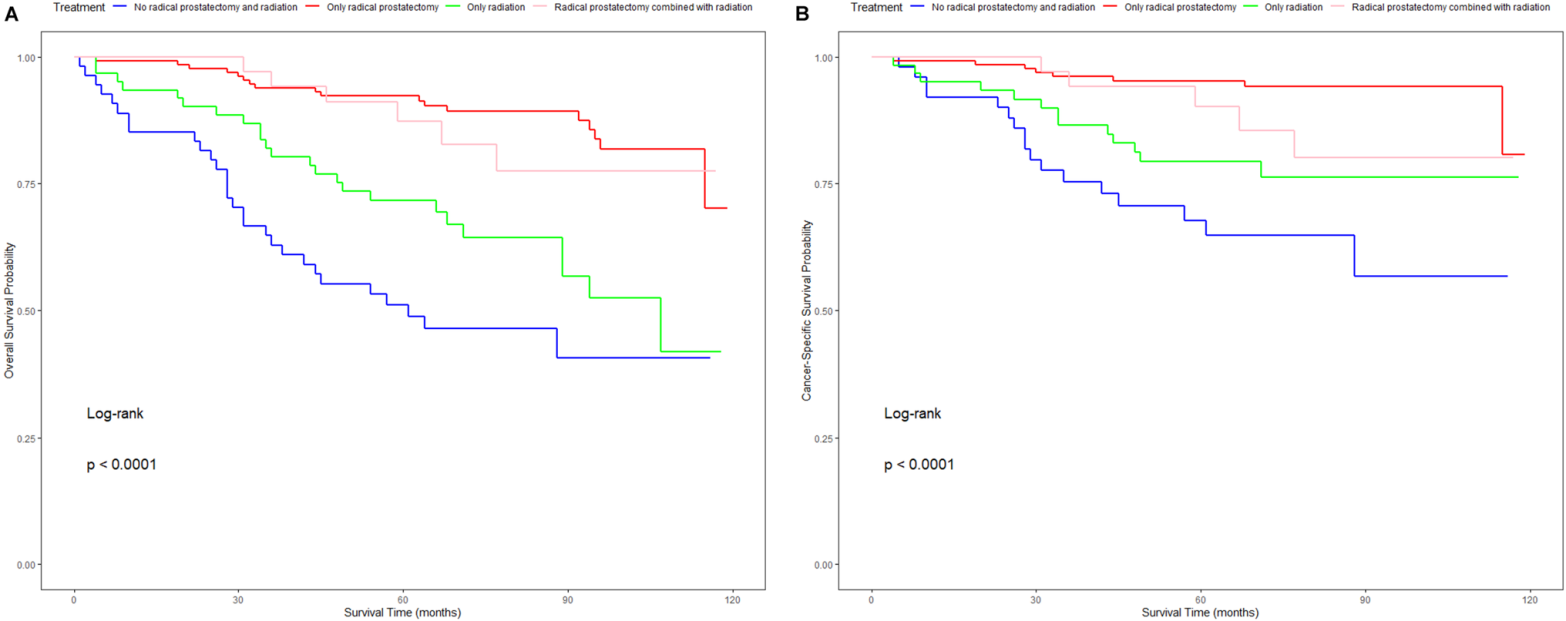
The Kaplan‒Meier survival curves show the different effects of different treatment on the OS (**A**) and CSS (**B**) rate of IDC-P patients

Kaplan‒Meier survival analysis was used to examine the OS and CSS of patients in various groups based on the findings of the multivariate analysis. The results of the Kaplan‒Meier survival curves were as follows: in terms of OS, patients in the localized group demonstrated a much better prognosis than other patients in various summary stages (*P < *0.01). The prognosis of patients with moderately and well-differentiated grades was considerably better than that of patients with poorly differentiated grades (*P < *0.01). In terms of CSS, the group with PSA levels demonstrated levels at 20 ng/ml with the worst prognosis, while the group with PSA levels between 4 and 10 ng/ml were considerably better than the other groups (*P < *0.01). The prognosis of patients who presented with lung or bone metastases was considerably worse than that of those who did not (*P < *0.01). Based on the summary stage, patients in the localized group had a considerably better prognosis than those in the regional and distant groups (*P < *0.01). According to the AJCC stage categorization, patients in Stages I and II had a better prognosis than those in Stages III and IV (*P < *0.01) (Figs. 1, 2). Meanwhile, we compared the survival prognosis of IDC-P patients in the radical prostatectomy and radiation group. IDC-P patients were grouped according to whether they received radical prostatectomy and radiation, and Kaplan‒Meier survival analysis was used to examine the OS and CSS of patients in various groups. In terms of OS, patients in the radical prostatectomy group had a much better prognosis (*P < *0.01), followed by the radical prostatectomy combined with radiation group, and the non-radical prostatectomy and non-radiation group had the worst prognosis. In terms of CSS, the prognosis of different groups was similar to the OS (*P < *0.01) (Fig. 3).

### Construction and validation of the nomogram prediction model

**Fig. 4:**
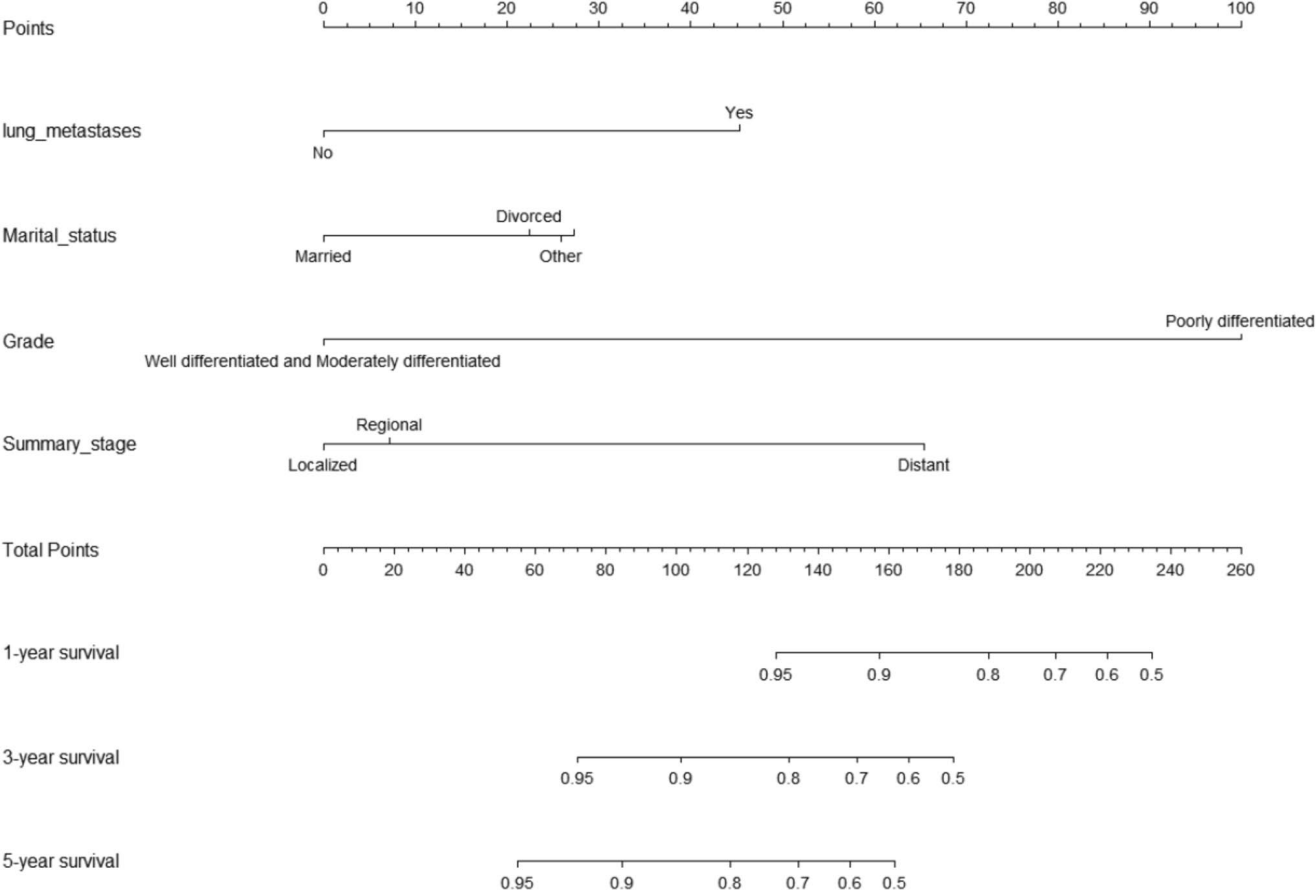
Nomogram predicting 1-, 3-, and 5-year OS for patients with IDC-P

**Fig. 5:**
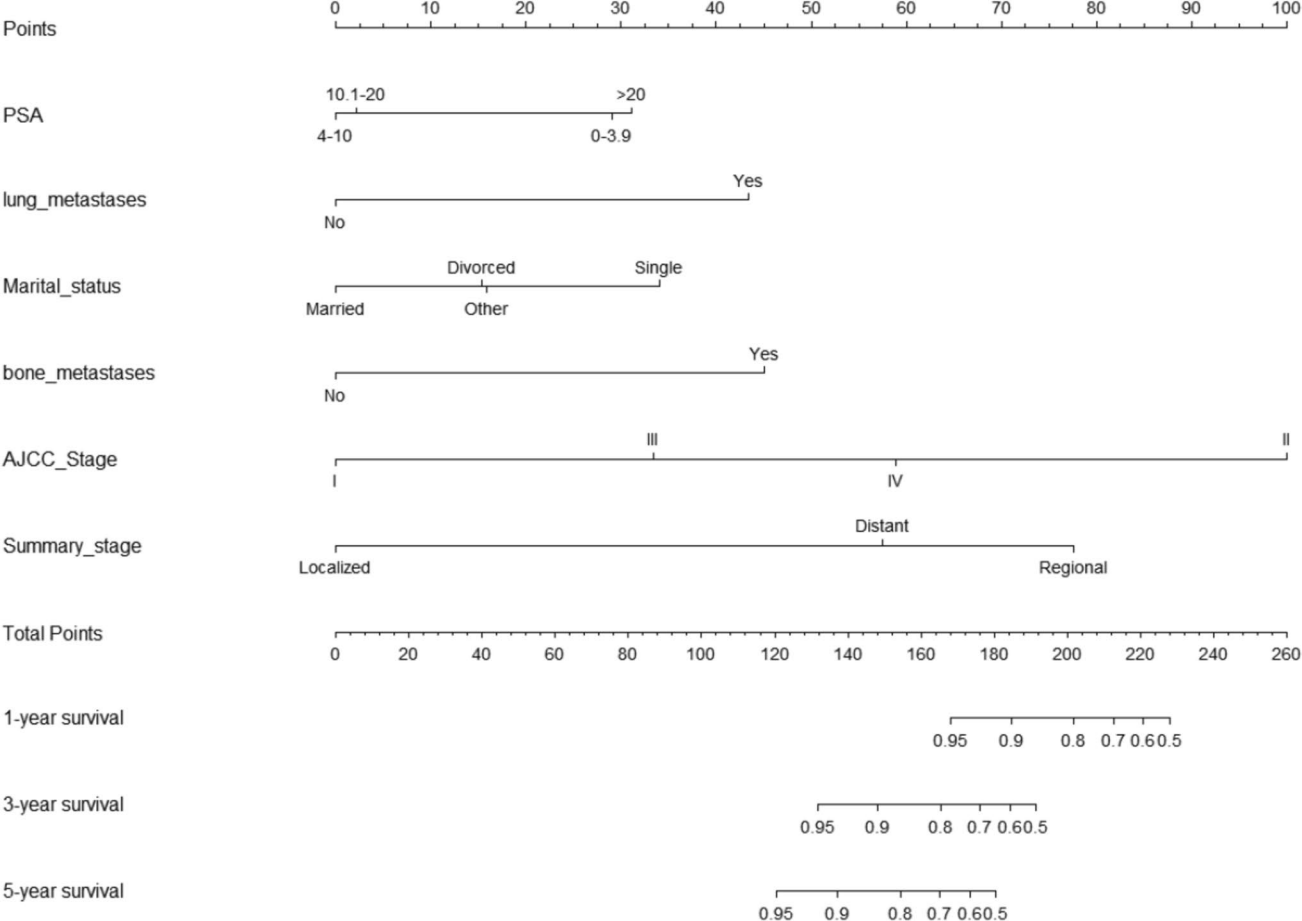
Nomogram predicting 1-, 3-, and 5-year CSS for patients with IDC-P

**Fig. 6:**
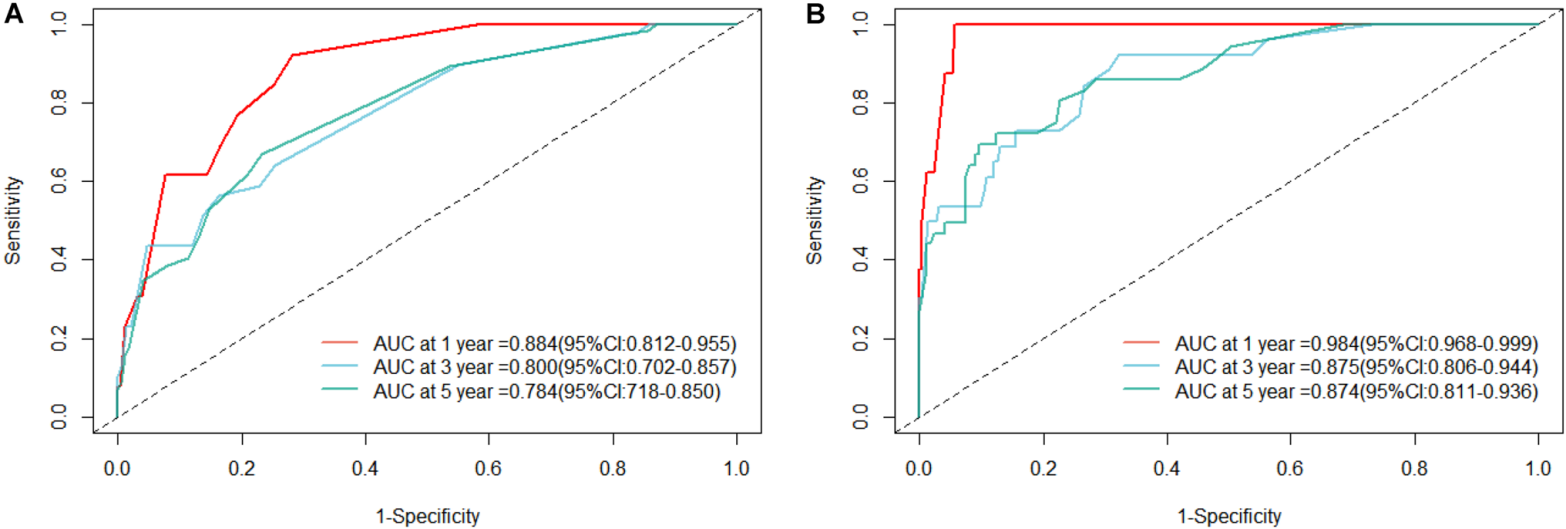
ROC of 1-, 3-, and 5-year of the OS for patients with IDC-P (**A**) and CSS for patients with IDC-P (**B**)

**Fig. 7:**
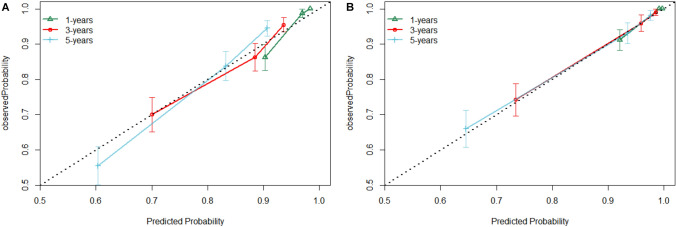
Calibration plots of the nomogram for predicting 1-, 3-, and 5-year OS (**A**)and CSS (**B**)

A nomogram was created for predicting OS prognosis using the four selected variables obtained from multivariate Cox regression analysis (Fig. 4). Using the six selected risk factors, a different nomogram was created to predict CSS prognosis (Fig. 5). These nomograms combined all the predictors and offered a visual tool for forecasting survival at 1, 3, and 5 years. The total score, which is calculated by adding the scores given to each variable, can be used to forecast the rate of survivors of patients at various time points. The bootstrap validation (1000 times) produced a C-index of 0.744 (95% CI 0.687–0.797), while the C-index for the OS risk prediction model in this study was 0.744 (95% CI 0.634–0.854). The bootstrap validation (1000 times) produced a C-index of 0.831 (95% CI 0.660–0.999), and the C-index for the CSS risk prediction model was 0.831 (95% CI 0.708–0.954). These findings show that both models can make a fair distinction between OS and non-OS patients as well as between CSS and non-CSS patients. ROC curves were produced for 1-, 3-, and 5-year OS and CSS to further demonstrate the model’s capacity for discrimination (Fig. 6). The model’s capacity to distinguish between favorable and unfavorable occurrences at various timepoints was assessed using AUC curves. For the 1-, 3-, and 5-year survival of OS and CSS patients, calibration curves were generated using the 1000 bootstrap resampling approach to evaluate the model calibration (Fig. 7). The calibration curves show that the projected survival probability and the actual survival probabilities observed agree. The model’s accuracy in predicting survival outcomes was demonstrated by the good alignment between the calibration curves and the ideal line.

**Fig. 8:**
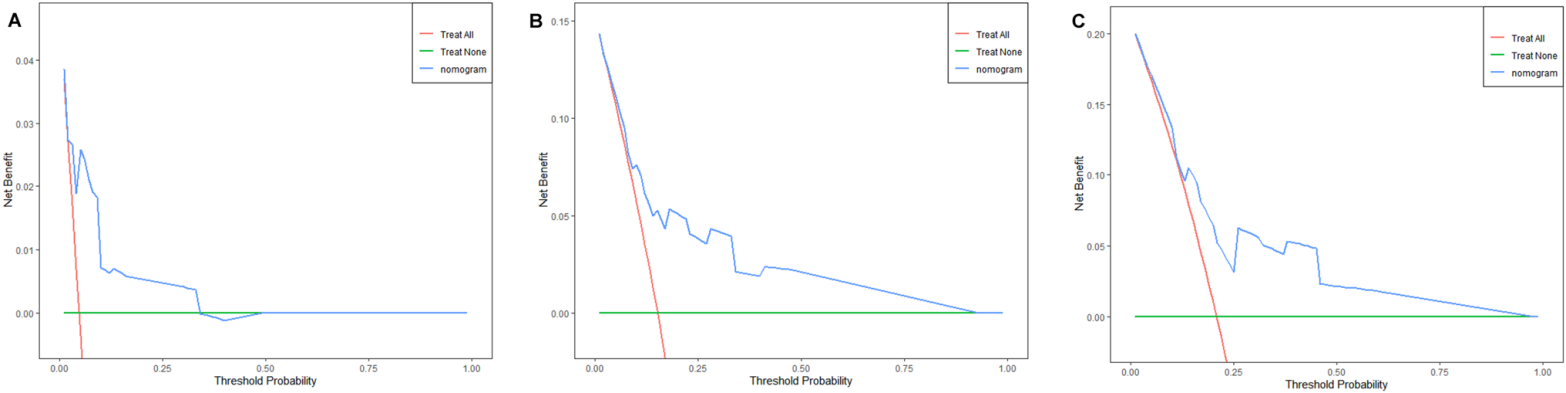
Decision curve analysis for evaluating the net benefit of nomogram for predicting 1-, 3-, and 5-year OS: **A** 1-year net benefit, **B** 3-year net benefit, and **C** 5-year net benefit

**Fig. 9:**
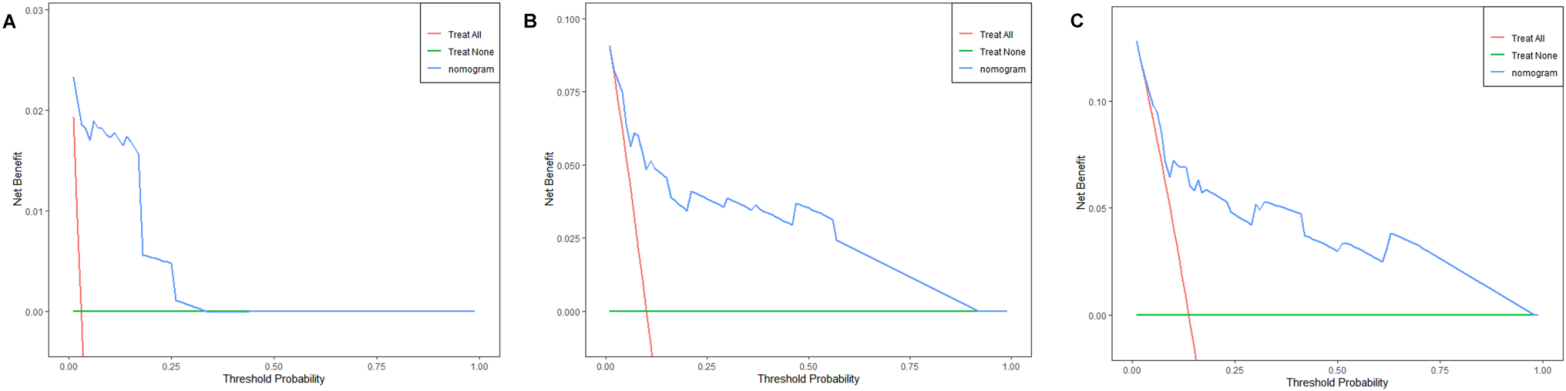
Decision curve analysis for evaluating the net benefit of nomogram for predicting 1-, 3-, and 5-year CSS.: **A** 1-year net benefit, **B** 3-year net benefit, and **C** 5-year net benefit

Beyond conventional diagnostic performance measurements such as sensitivity, specificity, and AUC, DCA is a useful technique for assessing the clinical value of a model by considering the preferences of patients or decision-makers in clinical practice. For the patients’ 1-, 3-, and 5-year survival outcomes in this study, DCA curves were created. The DCA curves consider various threshold probabilities to incorporate the clinical net benefit based on model prediction. The scenario represented by the green horizontal line has all patients classed as negative, yielding a net benefit of zero. The scenario where all patients are categorized as positive, resulting in a negative net benefit, is represented by the red diagonal line. The net advantage of the model is shown by the dashed line. At the 1-, 3-, and 5-year timepoints, the findings of the DCA curves revealed strong clinical net benefits, demonstrating the model’s high clinical applicability (Figs. 8 and 9). These results show that clinical decision-making can significantly improve patient survival outcomes by considering the model’s predictions.

## Discussion

IDC-P has some unique characteristics and features. First, IDC-P tends to occur in older men, especially those over the age of 60. Second, the cancer is usually slow-growing, with inconspicuous early symptoms, and is often detected at an advanced stage. This makes early diagnosis and treatment a major challenge. In addition, the pathologic features of IDC-P are of interest. The cancer originates from the ductal gland epithelial cells of the prostate and usually presents as glandular hyperplasia and heterogeneous hyperplasia. Globally, the clinical incidence of prostate cancer is increasing at a rate of 4% to 6% annually, making up 7.3% of all cancer cases combined. In more than half of the world’s nations, men are most frequently diagnosed (Sung et al. 2021). The second most common disease in men and the sixth major cause of cancer-related deaths worldwide is prostate cancer. A total of 359,000 fatalities and 1.276 million new cases were predicted to have occurred in 2018 (Culp et al. 2020). In the absence of aggressive prostate cancer, IDC-P is relatively uncommon but has a major impact (Robinson and Epstein 2010).

TNM staging, PSA, and Gleason score are currently the key parameters affecting the survival rate of prostate cancer. There is a need to investigate new trustworthy evaluation indicators or predictive tools because existing indicators are not yet able to appropriately measure patient survival. Compared to individual indicators, nomograms are often used as prognostic evaluation tools for cancer and can estimate the likelihood of specific occurrences for each individual by including several risk factors, offering a more precise estimation of patient prognosis (Balachandran et al. 2015; Feng et al. 2019). There is currently no large-scale data-based prognostic model for IDC-P. To thoroughly analyze the clinical traits and prognostic variables of IDC-P patients, we used the SEER database’s characteristics, which encompass a large sample size. We performed univariate and multivariate analyses of factors that could affect the prognosis of IDC-P patients using Cox regression analysis, thereby finding the key variables affecting prognosis. The purpose of this research is to contribute to the theoretical underpinnings for the prevention and treatment of IDC-P.

IDC-P is strongly correlated with aggressive prostate cancer according to research, and it is much more common in high-risk conditions (Porter et al. 2017). IDC-P is more likely to have higher pathological grades than other histological types, be more likely to develop distant metastases, have a worse prognosis, and die. Similar to high-risk adenocarcinoma, it has a favorable prognosis (Meeks et al. 2012; Morgan et al. 2010). IDC-P is regarded as a bad pathological subtype with a worse prognosis since it virtually invariably appears in high-grade tumor tissue. In radical prostatectomy, IDC-P is linked to unfavorable outcomes, including high Gleason scores, significant tumor volumes, and high pathological grades.

In addition, this study showed that grade is an independent predictor of OS in patients with IDC-P. In addition, it predicts clinical outcomes independently of other recognized clinical and pathological markers (Zhou 2018). After radical prostatectomy, the pathological type of IDC is a standalone predictor of progression-free survival (Cohen et al. 2000; Kimura et al. 2014; O'Brien et al. 2011; Rubin et al. 1998; Wilcox et al. 1998). In patients undergoing neoadjuvant hormone therapy, it is also a reliable indicator of biochemical recurrence (Kimura et al. 2014; Efstathiou et al. 2010; O'Brien et al. 2010). High Gleason scores, larger tumor volumes, and adverse prognostic factors such as extra prostatic invasion and seminal vesicle invasion are frequently linked to the existence of IDC-P. Without respect to the status of the treatment, IDC-P is linked to worse results (Divatia and Ro 2016).

In this study, 59.6% of the included IDC-P patients had a Gleason score of 7 or higher, indicating a link between IDC-P and high-grade differentiation. Detailed descriptions of the clinical features of IDC-P have sometimes been lacking in earlier investigations. Meeks et al. (2012) have outlined the characteristics of IDC-P; however, additional study is needed to determine its prognostic aspects. IDC-P has fewer lymph node metastases than acinar adenocarcinoma, according to research by Vignesh et al. (2015). Multifactorial Cox analysis showed that PSA was an independent predictor of CSS. IDC-P is linked to decreased PSA values, which might delay diagnosis and possibly increase the risk of metastasis, according to several studies. IDC-P patients have lower average PSA levels than acinar adenocarcinoma patients in the Gleason 8–10 range, while acinar adenocarcinoma patients have lower average PSA levels than IDC-P patients in the Gleason 6–7 range. Because of the development pattern of tumors in the prostatic ducts, which results in enhanced luminal PSA production and decreased serum PSA secretion, previous investigations have demonstrated that ductal carcinoma has dramatically lowered PSA secretion (Morgan et al. 2010). This study reveals that the total PSA level is concentrated at 4–10 ng/ml, indicating a typically lower PSA level, and that the overall rate of lymph node positivity in IDC-P patients is only 11.1%. We also observed an association between the summary stage and both OS and CSS in IDC-P. In addition, it was confirmed that individuals with a distant stage have a worse prognosis than those with a localized or regional stage. Summary stage is an important indicator for assessing the extent of tumor spread and clinical staging. Our findings showed that patients with late summary stages were more likely to be diagnosed with IDC-P. This may indicate that late diagnosis is related to factors such as tumor growth rate, pathological features, and patient behavior in seeking medical care. Late diagnosis may lead to delayed treatment and poor prognosis; therefore, our findings emphasize the importance of early diagnosis and screening.

In addition, AJCC stage is an important factor affecting patient CSS. Patients with early-stage disease have a better prognosis. A higher AJCC stage was linked to a shorter survival time and a worse prognosis, according to the Kaplan‒Meier survival curves. This study was significant since it introduced the first nomogram for forecasting clinical outcomes in IDC-P patients. IDC-P patients are more likely to present with advanced stages, such as T3 and T4, and a higher likelihood of metastatic illness, according to a study by Nithesh et al. (2021). IDC-P patients were primarily at the T2 (37.9%) and T3 (37.5%) stages when they were included in this study, indicating a higher malignancy grade and worse prognosis for this pathological type. Lung and bone metastases are common modes of distant metastasis in IDC-P and a marker of poor prognosis. These metastases result in significant OS and CSS survival. Multifactorial Cox analysis showed that lung metastases were an independent predictor of OS, and bone metastases versus lung metastases were independent predictors of CSS. Furthermore, IDC-P is known to spread to visceral organs, including the lungs and liver, as opposed to acinar adenocarcinoma, and instances of testicular or penile metastasis have also been documented (Samaratunga et al. 2010). IDC-P has been demonstrated to have greater invasiveness than pure acinar adenocarcinoma (Considine et al. 2021), which is compatible with the findings of this investigation. Interestingly, we observed an association between marital status and IDC-P. Married patients had better OS and CSS than unmarried patients. The study suggests that marital status may be associated with factors such as an individual’s lifestyle, social support, and mental health. Individuals with a stable marital status tend to have healthier lifestyles, such as regular exercise, healthy diet, and regular rest, which may help reduce the risk of prostate cancer. In addition, individuals in stable marriages often enjoy more social support, which may help them cope with stress and promote physical and mental health. Therefore, our findings support the association between marital status and prostate cancer.

The clinical importance of IDC-P in stereotactic body radiation (SBRT) for prostate cancer has been examined in various studies (Aizawa et al. 2022; Tom et al. 2019; Trinh et al. 2019; Van der Kwast et al. 2012) since 2012. The findings revealed a strong association between the presence of IDC-P and tumor metastasis, biochemical recurrence, and clinical recurrence following SBRT. According to a study by Trinh et al. (2019), the percentages of IDC-P-positive patients who exhibited biochemical recurrence following a period of up to 10 years were 29.6% and 64% in those who underwent adjuvant radiation therapy and those who did not, respectively. These findings imply that a more effective treatment strategy for locally advanced prostate cancer with IDC-P components may involve surgery in conjunction with adjuvant radiation therapy. Another study by Yu-Peng et al. (2017) found that although IDC-P patients were more likely to have adjuvant radiation therapy than acinar adenocarcinoma patients, they were less likely to undergo radical prostatectomy.

There is currently no consensus on whether patients with IDC-P should receive radical prostatectomy and radiation therapy; therefore, we performed a survival prognosis analysis of patients who receive radical prostatectomy and radiation. Overall, patients in the radical prostatectomy group in this study demonstrated a much better prognosis than the radical prostatectomy combined with radiation group, which may be related to the threat of intestinal toxicity associated with radiotherapy (Mazariego et al. 2020), which generally has a higher cost. Notably, radiation, surgery type, and systemic therapy were not identified as independent predictors in the multifactorial COX analysis, and furthermore, the reasons for patients who did not receive radical prostatectomy and radiation are unknown, all of which need to be further explored in future studies. Because standard treatment protocols for patients with IDC-P have not yet been established, current research is divided on whether to receive systemic therapy. Systemic endocrine therapy was considered ineffective in the early stages (Bates and Thornton 1973); however, Orihuela and Green (2008) reported that radical radiotherapy combined with androgen deprivation therapy resulted in better survival in patients with DA. Studies have shown that IDC-P may contain cancer cells capable of resisting androgen deprivation (Porter et al. 2018), leading to speculation that IDC-P may be resistant to therapeutic regimens for the treatment of aggressive prostate cancer. Regardless of the underlying mechanisms, these findings suggest that multimodal and novel therapeutic options are needed to treat IDC-P.

Taken together, these results provide some insights that can be applied in clinical practice. First, physicians and health professionals should emphasize patients’ marital status and consider this factor when assessing patient risk. Second, early diagnosis and screening are critical to the treatment and prognosis of prostate cancer, and physicians should actively promote relevant screening programs and awareness campaigns. Finally, our findings also draw attention to the importance of lifestyle and social support for the prevention of prostate cancer, and individuals should pay attention to maintaining a healthy lifestyle and establishing a good social support network. Future studies should further explore the mechanisms of these associations and adopt more rigorous study designs to validate our findings.

This study, however, has some limitations. First, as it is a retrospective study based on the SEER public database, some patients’ data may be missing, which could affect the analysis’s findings. Second, the database lacks data on the general health status of the patients, such as comorbidities, and the surgical expertise of the surgeons, both of which may affect the prognosis of the patient. The inclusion of patients with incomplete data and the small sample size in the SEER data could affect the study’s findings. To provide more solid support for the findings of this study, we recommend that future studies adopt a prospective design and incorporate more potential influencing factors for analysis. It is also recommended that sample sizes be expanded and multi-center studies be conducted in different regions to improve the external validity of the findings.

## Conclusion

The results of this study indicate that IDC-P have distinct clinical and pathological characteristics and a worsening prognosis. The results of the study also show that marital status, summary stage, grade stage, and the presence of lung metastases are significant factors influencing the prognosis for OS, while marital status, summary stage, AJCC stage, the presence of lung metastases, the presence of bone metastases, and PSA are significant factors influencing the prognosis for CSS. The created nomogram prediction model increases the precision of clinical decision-making by accurately predicting the prognosis of IDC-P patients. The nomogram prediction model will eventually require additional patient data for validation.

## Data Availability

Publicly available datasets were analyzed in this study. This data can be found here: https://seer.cancer.gov.
